# FAIR data – the photon and neutron communities move together towards open science

**DOI:** 10.1107/S2052252524011941

**Published:** 2025-01-01

**Authors:** B. M. Murphy, A. Götz, C. Gutt, C. McGuinness, H. M. Rønnow, A. Schneidewind, S. Deledda, U. Pietsch

**Affiliations:** a Institute of Experimental and Applied Physics Kiel University Leibnizstr. 19 24118Kiel Germany; b Ruprecht-Haensel Laboratory Deutsche Elektronen-Synchrotron DESY Hamburg Germany; c European Synchrotron Radiation Facility Grenoble France; d Department of Physics University of Siegen Siegen Germany; e School of Physics Trinity College Dublin Dublin2 Ireland; f Institute of Physics École Polytechnique Fédérale de Lausanne (EPFL) 1015Lausanne Switzerland; g JCNS at MLZ Forschungszentrum Jülich Jülich Germany; h Institute for Energy Technology (IFE) Kjeller Norway; SPring-8, Japan

**Keywords:** FAIR principles, photon and neutron sources, user organizations, open science, data storage, data pipelines, research infrastructures

## Abstract

The topic of data storage, traceability, and data use and reuse in the years following experiments is becoming an important topic in Europe and across the world. Many scientific communities are striving to create open data by the FAIR principles. This is a requirement from the European Commission for EU-funded projects and experiments at EU-funded research infrastructures (RIs) and from many national funding agencies. This is challenging for users of large-scale RIs such as neutron, photon, synchrotron and free-electron laser facilities. Users of photon and neutron (PaN) RIs employ a wide range of scattering, imaging and spectroscopic methods investigating the behaviour of matter with a broad scientific base across physics, chemistry and biology, including engineering, environmental, cultural heritage and medical applications. They produce large data volumes of up to 1 PByte per day in some cases. To ensure all these data are FAIR requires an enormous effort from PaN RIs. It requires not only the expansion of data storage capacity, but also the development and deployment of software for effective data storage, metadata schemes and implementation of effective data pipelines at each individual experiment across RIs. FAIR data also affect the carbon footprint related to large amounts of data and raise questions related to user authentication, rights of access and cyber security. The RIs alone cannot achieve such a transformational process. For successful open science, cooperation of the user communities is essential as they need to create and utilize existing tools to deliver FAIR data. In this white paper, the European PaN community outline and discuss the role and responsibilities of the users and RIs and their common accountability to achieve FAIR data. This paper shall serve as a starting point for a common user and RI approach on the European scale to achieve FAIR data.

## Abstract 2

The German project DAPHNE4NFDI together with the European Synchrotron and Free Electron Laser User Organization (ESUO), the European Neutron Scattering Association (ENSA), and European synchrotron and neutron facilities continue the development of FAIR data handling procedures laid out by the EU-funded projects PaNOSC and ExPaNDS. Written by members of these organizations, this ‘white paper’ documents the current status of this discussion.

## 1. Introduction: photon and neutron large-scale facilities and their user community representation

The process of direct FAIR implementation in photon and neutron (PaN) research infrastructures (RIs) is guided by LEAPS (League of European Accelerator-based Photon Sources) and LENS (League of advanced European Neutron Sources), whereas the process on the side of European users is moderated by ESUO (European Synchrotron and Free Electron Lasers User Organization) and ENSA (European Neutron Scattering Association), headed by respective executive boards. In Germany, the National Research Data Infrastructure (NFDI) is intended to systematically develop, sustainably secure and make accessible the data holdings of science and research and to network them (inter)nationally. The DAPHNE4NFDI consortium (Barty *et al.*, 2023 ▸) brings together the German PaN user representatives via the KFS (Komitee Forschung mit Synchrotronstrahlung) and KFN (Komitee Forschung mit Neutronen) and creates a funded framework to work together with RIs to achieve the common aim of FAIR open data. These activities are now coordinated with ESUO and ENSA.

The aim of this paper is to summarize the current status of implementation of FAIR principles in PaN RIs and communities and to make a proposal of how the process can be accelerated and streamlined in the future. A first draft prepared by members of the DAPHNE4NFDI, ESUO and ENSA communities was presented to delegates at a common ESUO–ENSA–DAPHNE4NFDI workshop held at PSI in November 2023, initiating an intense discussion and suggestions for improvement of the document. The current version is based on this extensive discussion of open data principles and their impact on each user experiment. This document intends to outline best practice and responsibilities with the aim to motivate a large number of European users to contribute to the development and to make best use of FAIR principles.

## 2. FAIR data principles, risks and benefits to the community

In order to understand the cost, risks and benefits to the community, it is first required that we define FAIR data. The definitions shown in Table 1 ▸ are taken from Wilkinson *et al.* (2016 ▸). Following the publication *The FAIR guiding principles for scientific data management and stewardship*, the FAIR principles mean that all data are *findable*, *accessible*, *interoperable* and *reusable* across research RIs and scientific domains. This concerns creation, storage and further use of any data recorded during an experiment. In detail, data need:

*To be findable.* Metadata (see below) and data created by users should be easy for humans and computers to find. Machine-readable metadata are essential for the automated discovery of records and services.

*To be accessible.* Once the user has found the data they want, they need to know how to access it, possibly involving authentication and authorization.

*To be interoperable.* The data usually need to be integrated with other data. In addition, the data must work with applications or workflows for analysis, storage and processing.

*To be reusable.* The goal of FAIR is to optimize the reuse of data. To achieve this, metadata and data should be well described so that they can be replicated and/or combined in different environments.

Further specifications of the FAIR guiding principles are listed in Table 1 ▸.

Historically, the forerunner of a FAIR data repository had been instigated via the Cambridge Crystallographic Data Centre (CCDC) through the Cambridge Structural Database (CSD), a not-for-profit organization with charitable status, established in 1965 with model coordinates then later improved with digital storage for the addition of structure factors. Although it was initially open, the access mode to CSD data later changed to subscription. However, the stored structure factors already allow for a reproducibility check of any subsequent study. The distinction between reproducibility and replicability was clarified in the 2019 US National Academies Report (National Academies of Sciences, Engineering and Medicine, 2019 ▸). FAIR allows for both reproducibility and replicability when more challenging investigations are warranted. A paper from the IUCr Diffraction Data Deposition Working Group (DDDWG, 2011–2017) and now the IUCr Committee on Data (CommDat, since 2017) advocating various relevant aspects of raw diffraction data reuse has been published recently (Helliwell *et al.*, 2024 ▸).

The FAIR guidelines have been expanded and specified in more detail since their initial definition. For its implementation, a careful definition of metadata is of particular importance. Metadata are ‘data that provide information about other data’ (NISO, 2017 ▸), but not the content of the data (*e.g.* the text of a message or the image itself). Metadata should be *descriptive and structured* containing *administration* information describing *technical, preservation and rights issues.* Often a markup language is used for tagging semantic features. There are currently several approaches for how metadata can be defined, however, there is no generalized definition to date. Further discussion among users is required to find an approach that satisfies a large number of experiments and to establish a common definition library.

The cost of creating truly FAIR datasets is immense in terms of time and money, not only to learn and apply the principles, but also to develop and implement appropriate user-friendly tools. This requires an investment by both RIs and users. It is important that such tools and development thereof are sustainable, as well as being easy to deploy and maintain in the future. The barrier to adoption by users and, in particular, new and inexperienced users, should be as low as possible.

The risks can be viewed from two sides, one being the limited effectiveness of reaching a FAIR data platform despite best efforts due to the rich and complicated range of experiments carried out across the community. This risk needs to be mitigated through cooperation across the communities and RIs but requires a financial basis for development and maintenance of the tools as well as for data storage and curation.

The benefits of FAIR data are immense. In general, open data result in better science, starting with ease of data handling within a single research group or across a large cooperation for data analysis, to sharing data with other unrelated scientists who may re-analyse the data in order to extend understanding or even to make new original discoveries. Following the FAIR principles, data are always findable and available, even after the person who carried out the original measurement moves on. Clear documentation, the availability of the data and respective analysis tools lower barriers for reuse by others. A further benefit of having a clear and open data description is that it can be used for machine-learning and ‘digital twinning’ applications, allowing us to extract even the smallest details and make new predictions from a large data lake. Further, the regions who may benefit most from open and FAIR data may well be those that do not currently have PaN RI facilities, both within and beyond Europe.

Implementation of FAIR principles will have a high impact not only on planning and execution of experiments, but also on the structure and optimum selection of data worth depositing. Thus, traditional avenues of recording experimental workflows have to be defined and implemented for old and new experiments. As is the case in best scientific practice for planning and preparation of each experiment, it is necessary first to identify the scientific questions to be addressed. The next step is to identify the system to be investigated and to choose the appropriate method and sample environment. Documenting these steps with appropriate metadata is clearly beneficial. For the experiment, it is important to consider which variables are required and if they are independent or dependent variables, *i.e.* what will be varied and what will and should remain constant during the measurement. Considering this key information will impact the structure of data and definition of metadata. Careful definition of data/metadata provides a solid basis for the data pipeline, in particular for data reduction and analysis, and allows interoperability and reuse. In contrast, a less-than-careful definition of required data and metadata creates a second risk that later essential data are missing, or that measured data are not rich enough to accurately describe the dataset. In the worst case this can mean that the data can no longer be assigned to a certain sample or sample run and that this dataset thus may lose its scientific value. This risk is reduced by writing an instructive logbook interlaced with the beamline log and the metadata catalogue. Not only individuals but also the entire community can benefit from consideration of FAIR principles. Exchange and reuse of data are simplified if many users follow the same structure for their data/metadata.

## 3. Role and responsibilities of research facilities

Large-scale RIs are a central point of contact for users where motivation, information, knowledge and skills can be transferred to user groups visiting for experiments. Many users would like to have guidelines on how to prepare before the experiment so that they will be able to create a FAIR experiment. The RIs can illustrate best practices in data handling and can train users in the use of the most modern tools and applications for creating and maintaining FAIR and open data practices in a sustainable manner. In the process of implementation of FAIR principles, RIs should have a general data policy following the FAIR principles, which is continuously discussed and updated with the user community. This concerns many aspects including acquisition and storage of raw data and metadata during experiments, reduction of raw data, accessibility to data, data security, and embargo time.

In the process of implementation of the FAIR principles, users expect that each large-scale facility will develop experiments to follow FAIR principles. This means that each experiment (beamline) captures data and metadata considering a ‘general’ metadata catalogue, defines the parameters to be listed in the experimental logbook, enables raw data storage preferentially in a reduced or compressed manner (data compression workflow) and provides a certain number of analysis tools covering the majority of experiments. To make this process effective and attractive for users, the format of captured data and metadata should be as uniform as possible and follow similar general structures not only among the beamlines of a single facility, but also across a large number of PaN facilities. Specifically, metadata related to the instrument and its infrastructure should be provided by the facility. Users can contribute to the process of unification by clear communication while carrying out experiments at different PaN facilities. User-adapted analysis tools should be integrated into the analysis pool of the particular beamline and made available for others. The procedure of data access should be common for all RIs. This means the RIs provide the computer power and software to store that data for 10 years to allow (re)-analysis of the stored data. RI facilities implement permission-based access to data during and after the embargo period and provide a tool to generate bespoke digital object identifiers (DOIs) for raw data and data used in publications. The PaN facilities guarantee an embargo period of 3 years extendable by demand from the responsible user group. Finally, facilities should communicate an estimate of the CO_2_ equivalent consumed to create and store the data.

## 4. Role and responsibility of users

**Figure 1:**
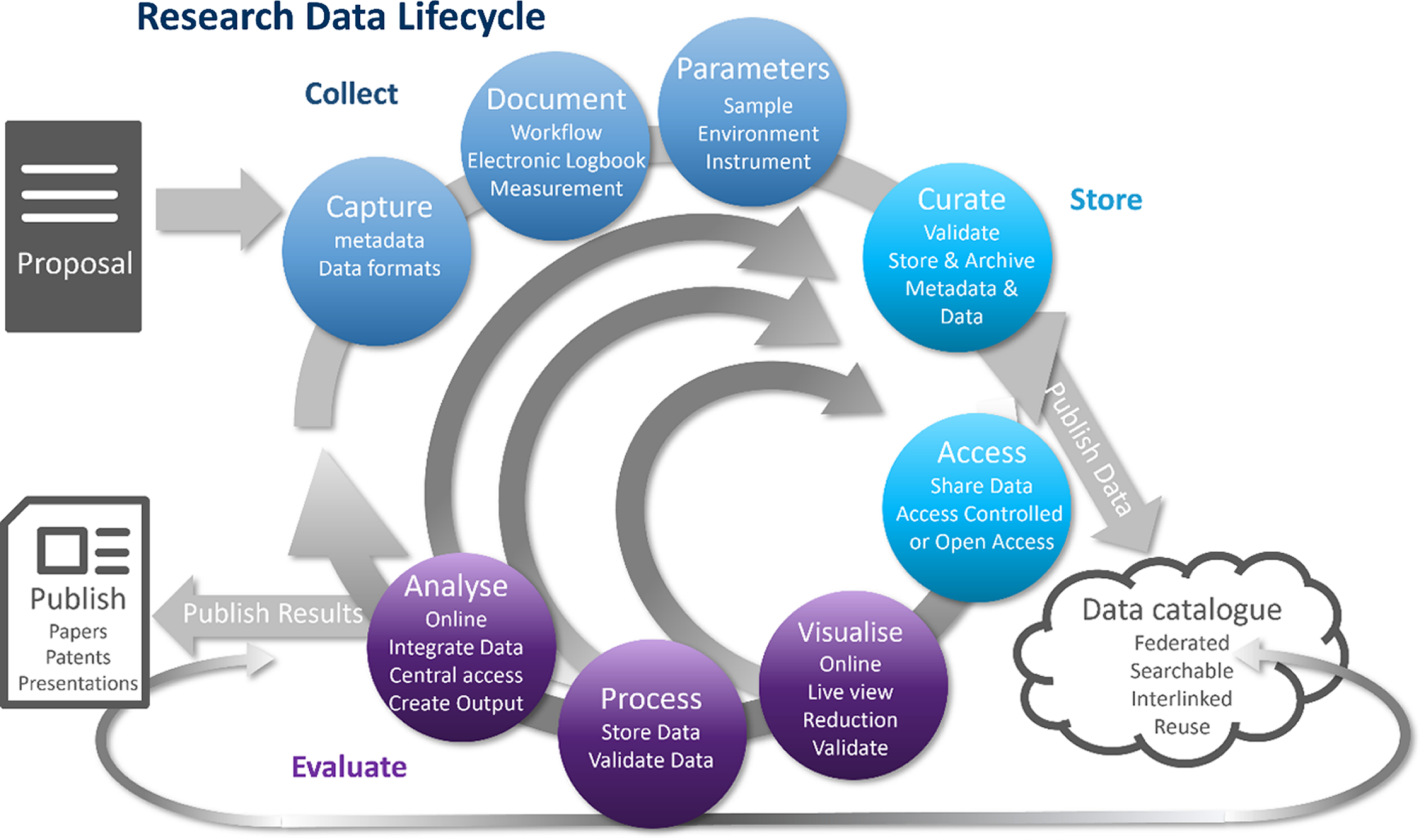
Research data lifecycle of FAIR data (ExPaNDS/PaNOSC, 2020).

Users of PaN facilities have a unique possibility to interact with RIs and amongst each other at a central point to create a common set of standards and tools required for a FAIR data research data lifecycle (Fig. 1 ▸) that is appropriate and robust. It is important that they engage with the facility and provide accurate information throughout the application, assessment and execution of the experiment including analysis during and after the experiment. The RIs can inform the users about best scientific practices and lead the way by providing the necessary tools for implementation. However, a scientifically useful implementation of the FAIR principles also needs a strong engagement of the users in the process of design, preparation and execution of an experiment at a PaN facility.

Users are specifically responsible for performing experiments (with varying levels of help from the instrument scientist), collecting data and ensuring the scientific quality of the data. Good scientific practice also entails that they provide correct and comprehensive metadata including sample information and a full description of analysis methods used to interpret the data. They are required to provide clear descriptive experimental proposals and deliver informative experimental reports. Related publications should follow the principle of best scientific practice, citing the DOI for the published data archived in the data repository and the experiment workflow and analysis pipeline. Here each author should have an ORCID identifier. The ongoing development of best practices should be included in the education of young researchers as an integral part of good scientific practice.

The main steps of the data lifecycle for facility users are shown in Fig. 1 ▸. It starts with the proposal describing the proper design of the experiment, considering the required sample environment, but also the optimum selection of parameters worth recording and depositing. While collecting data by an angular scan or recording a detector frame, for example (Fig. 1 ▸), the users ensure that metadata and other documents such as electronic logbooks record this other information and experiment descriptors which are not automatically captured by the RI during the experiment, and that these are as complete and correct as possible. These data are stored and archived. Here a proper sample identifier must be defined and listed in both the experimental logbook and in the metadata catalogue. Based on unified metadata schema, users need to define their specific metadata and provide good metadata for storage. This includes a description of the sample workflow including the history of how the sample was prepared for the experiment. It is important to capture the experimental workflow (experimental steps performed: sample cell descriptors if it is a user-designed cell, motor positions, specific runs and so on). Here, it is useful to indicate which data measured are worth storing (true results) or which data can be deleted because they are either related to, establish or verify the experimental setup, or are erroneous runs. This first curation of data and choice of deletion must also be visible in the metadata. Figures extracted from the raw data are also worth storing. During or after the experiment, users start to analyse data, either to verify expectation or to find out if the experimental parameters are or were properly selected to obtain the best data. Results of this analysis workflow need to be documented and the results need to be stored either in logbooks or as analysed data as the last step in the data curation. Finally, a digital object identifier (experiment DOI) for publication of relevant data and for the analysis pipeline has to be created and maintained.

In order to define best practices, the community needs to define the data formats (NeXus/HDF5) and develop a descriptive ontology of metadata based on community and facility standards as well as procedures for capturing the experimental workflow during an experiment or measurement. Experimental protocols and logs are considered essential for reproducibility of science. Logbooks and descriptions of the experimental workflow and of the analysis methods should be linked to the relevant experimental data. Analysis code should be openly available and usable at the beamline and from elsewhere. Acknowledgement of authorship is important and must be recorded in the metadata catalogue.

## 5. Shared accountability and workflows and best practice procedures

Users and RIs work together to achieve a complete FAIR data scheme ranging from the experimental conception, sample preparation, carrying out the experiment, and data analysis for the interpretation and publication of a scientific paper in a peer-reviewed journal. Defining standards and deciding which information such as data metadata, workflow, sample-persistent identifier PID, software DOI *etc.* are important and require dialogue and exchange between RIs and their users. Here, the user representatives at a facility, or at national or international level (*e.g.* ESUO, ENSA), can be an informed voice for the users.

RIs and users need to provide constructive input and feedback for setting up tools and procedures that enable FAIR data management. Both are responsible to check and monitor that procedures function well and should work on further improvements together. A bilateral involvement in the process allows an agile development that the community as a whole can benefit from in the future. In detail, this means that both users and RIs work together on scientific data policies. They should form organizations (*e.g.* user organizations, combined working groups or similar) that guarantee that data handling aiming towards FAIR data is constantly developed and continues to receive input from the communities as a whole. Moreover, users and RIs together ensure that scientific good practices are continually followed. This requires a proper definition of standards. For the process of implementation, common basic definitions of metadata and implementation of analysis workflows are necessary in the near future. Finally, since resources are not endless, a dialogue between user communities, RIs and funding agencies is required to decide which data should be kept in which storage format and for how long

## 6. Current status of FAIR open data implementation at the PAN facilities

To meet the challenges given by the ever-increasing volumes of data and by data fragmentation, in 2015, the European Commission (EC) proposed a new concept: the European Open Science Cloud (EOSC). It aims to provide scientists with access to data, software and services from as many scientific data sources in Europe as possible, by making them FAIR across research RIs and scientific domains. To achieve this, the EC financed a large number of projects to kickstart the building of the EOSC in many domains where the Photon and Neutron Open Science Cloud (PaNOSC, 2018–2022) and ExPaNDS (2018–2023) projects were executed as seed projects for the majority of European PaN RIs. The main goal of the project was to address the FAIR principles in the PaN RIs by equipping them with all the necessary software, policies, and required legal and administrative frameworks. Throughout its implementation, PaNOSC has provided common policies, strategies and solutions for enabling open science through the adoption of FAIR principles across three European PaN RIs and three European Research Infrastructure Consortia (ERICs), helping to make their data open and available to the EOSC. PaNOSC and ExPaNDS have paved the way to make data produced at PaN RIs across Europe easily accessible: curation of data has started and the data are made available under an open FAIR data policy.

Overall, all RIs have adopted, or are in the process of adopting, the PaNOSC FAIR research data policy framework, which serves as guidance for FAIR data stewardship (Carboni, 2022 ▸). It does this by the generation of (meta)data from raw data recorded from each experiment, to analysis, through to publication and reuse. For this purpose, PaNOSC implemented effective data management plans across PaN RIs to ensure that RI users and support teams are aware of the data volumes that will be produced and how to process them throughout the whole data lifecycle. Such information is also beneficial to forecast the IT infrastructure required to support an experimental programme based on a more detailed understanding of user needs. To make PaN data easily findable and accessible across a multitude of PaN RIs in Europe, a federated search application programming interface or PaN data catalogues and a common protocol for harvesting data and metadata have been developed to make public datasets available to third-party EOSC cross-discipline repositories. This service provides a unified approach across RIs for PaN scientists to find, filter and score/rank datasets and publications from any number of configured sites based on relevant domain-specific metadata from any facility for any experiment where the embargo period has ended.

As a further achievement of PaNOSC, a community metadata standard for PaN sources (NeXus/HDF5) is in the process of being adopted to make data interoperable and reusable. Electronic logbooks have been developed at a number of RIs to capture what happens during experiments and keep track of instrument settings for future usage. Some facilities such as the ESRF already generate DOIs for each experiment, allowing one or more datasets to be cited in publications. In the future, experimental DOIs will be provided by each PaN facility either automatically or by request. To further lower the barriers to reuse of open data, users should be enabled to explore data through their web browser after identifying a dataset of interest. To this aim, PaNOSC has provided a remote access viewer infrastructure to enable and contain FAIR data services for users of the PaN community, and later on for scientists from across domain borders, through the EOSC, at individual experiments. This has been achieved by developing and making available two types of data analysis services: remote desktops for graphical software use and Jupyter Notebooks (https://jupyter.org/) for recording data analysis routines. These applications run on virtual machines and can be accessed remotely via the open-source data analysis online portal. For example, the *VISA* (*Virtual Infrastructure for Scientific Analysis*) platform, (https://bit.ly/VISA-video), as part of the Virtual Neutron and X-ray Laboratory (ViNYL), offers remote control, simulation services of pilot experiments, experimental setups and data analysis procedures. In fact, *VISA* is a new way for academic and industrial researchers to access data and access advanced analysis tools from anywhere.

Finally, PaNOSC and ExPaNDS have developed an e-learning platform, (https://e-learning.pan-training.eu), hosting free education and training for scientists and students, with online interactive courses on both the theory of PaN science and how to use Python code or software for data reduction and modelling. Moreover, a training catalogue for PaN science allows browsing instructional material and resources from institutes around Europe.

## 7. Current status of FAIR open data implementation by PAN users

Since both PaNOSC and ExPaNDS projects have expired, the members of the two projects formed the so-called PaNOSC collaboration. The PaNOSC collaboration represents all photon and neutron sources in Europe in the EOSC with the aim to implement PaNOSC and ExPaNDS achievements in all PaN facilities. To further develop EOSC principles at PaN RIs and to make them sustainable, LEAPS has proposed a road map to continue the work started in PaNOSC and ExPaNDS and extend its impact to the users of LEAPS and LENS RIs as well as the scientific community and society in general over the next 7 years, (Götz & Phys, 2023 ▸). However, the true implementation has to be realized by each facility considering national conditions. Here, users should give a clear signal that they want to have equal and good conditions at all PaN facilities.

In addition, EU funding of EOSC projects continues. This is possible via the ‘OSCARS’ project and the OSCARS Open Calls starting in January 2024 with the aim to support cross domain collaborations to promote open science in the community (https://science-clusters.eu/).

In Germany the DAPHNE4NFDI consortia, financed through the Deutsche Forschungsgemeinschaft: National Research Data Infrastructure (NFDI) programme, is aiming to create a comprehensive infrastructure to process research data from PaN RIs according to the FAIR principles. DAPHNE4NFDI brings together users representing key scientific application domains with the large-scale research facilities in PaN science to advance the state of data management in the community. Key objectives within DAPHNE4NFDI are: to improve metadata capture through consistent workflows at RIs supported by user-driven online logbooks that are linked to the data collection; to establish community repositories of processed data, new reference databases and analysis code for published results, linked, where possible, to raw data sources in order to sustainably improve access to research data; and to develop, curate and deploy user-developed analysis software on facility computing infrastructure through common data analysis portals. DAPHNE4NFDI aims to have impact across the wider European and international PaN community. Importantly, the NFDI strives to connect different scientific consortia opening FAIR PaN data to scientists in related fields such as material sciences, physics, biology, engineering *etc.* who are not users of RIs but who are interested in the scientific data produced. In this way, the data of the PaN community will have a much larger scientific impact beyond our community.

## 8. Sustainability and carbon footprint of FAIR data

FAIR data help to reduce the carbon footprint of our science by avoiding repeating experiments and by optimizing the reuse of existing data. This is especially important due to the high energy consumption of large-scale infrastructures such as neutron sources, synchrotrons and XFELs resulting in a considerable carbon footprint for each experiment. However, storing data also requires considerable curation efforts and also energy. Avenues on how to compromise between storing all raw data or only processed data or only data flagged for publication need to be explored. This requires active discussion within the community. It also requires balancing the amount of data readily available in the cloud versus data more permanently stored on tape, as the energy consumption of data in the cloud is much higher than tape storge.

A possible long-term solution would be the establishment of a common data repository for the PaNs which, if set up in consultation with users and RIs, could have several benefits. First, it could optimize the technology of data storage (hardware); second, it would help standardize data and metadata formats; third, it would redistribute the cost and the burden of data archiving from the individual PaN facilities around Europe; fourth, it could reduce the running costs and carbon footprint of data archiving; fifth, data download (*i.e.* availability) could be via a single entry point. A possible implementation of this could be the creation of a so-called EOSC Node for the PaNs. The concept of EOSC Nodes is under discussion between the EOSC Association, the EC and member states as the way forward for the EOSC as a federation of nodes. The proposal for the PaNOSC collaboration to study the implications of becoming the PaN EOSC Node is endorsed by the LEAPS Chairs. Once the EOSC Node concept is mature, it will be presented to and discussed by the representatives of LEAPS and LENS as a possible way forward.

Finally, reprocessing large amounts of data is also connected with energy consumption and the user community needs to develop an active awareness that the ‘reproducibility’ in the slogan FAIR is not free of cost but also comes with an energy price. Optimizing algorithms, balancing knowledge gain versus costs for reprocessing and minimizing the energy consumption in data treatment should become a central part of the future development of FAIR data practices (*Resource-aware Research on Universe and Matter: Call-to-Action in Digital Transformation* – white paper in preparation).

## 9. Opportunities for transformation within the user community: a perspective for users and research facilities

Introducing and implementing this transformation to FAIR and open data will help to increase the transparency in our scientific discipline. Sharing data and workflows on this new level will also help to increase exchange and discussions across different scientific disciplines. FAIR data will provide new possibilities to share results across collaborations and far beyond into the scientific community, and even enable citizen science. Based on FAIR documentation, data can be reused long after publication allowing eventual new interpretations in the future. All of these steps together will improve the quality of our science, paving the road for new breakthroughs by re-using FAIR PaN data. For example, a review and analysis of SARS-CoV-2 crystallography data in PaN and PDB repositories obtained since 2020 has enabled new interpretation of roles of proteins in coronavirus function (Fyfe *et al.* 2023 ▸; Kandler *et al.*, 2023 ▸).

These benefits, however, can only become reality if a large number of PaN users and PaN RIs can be convinced to follow FAIR data management. This in turn requires collaborative approaches, low access barriers and easy-to-use tools for FAIR data. It also requires disseminating knowledge about FAIR and FAIR data management to the next generation of scientists, ensuring their place in good scientific practice. Finally, we need to be aware that making data FAIR is a process which continuously develops parallel to the developments of experimental setups, sources, detectors *etc.* Here long-term commitments from RIs, users and funding agencies are needed to ensure sustainable solutions beyond a single generation of PhD students.

In summary, implementation and application of FAIR principles is a big step towards open science. Because research at PaN facilities is financed by public money, the research results are not private and belong to the public. Moreover, considering the considerable costs of each experiment, reuse of the same data by other groups might be desirable. Reuse of data also becomes attractive as access to large facilities is limited due to the high competition of high-ranking proposals or intentional abstinence of travelling to reduce the carbon footprint, or simply by missing funding for personal access. The latter might be important even for countries without their own PaN facilities. However, reuse is only possible if FAIR principles are followed, which requires engagement of all research groups. In this paper we have summarized the current status of implementation of FAIR principles, and the responsibilities of PaN facilities and the users. In conclusion, the main mission of this paper is to convince all parties about the importance and use of FAIR principles. The user organizations, ESUO and ENSA as well as DAPHNE4NFDI, will provide maximum support.

## Figures and Tables

**Table 1:** Detailed description of FAIR data (Wilkinson *et al.*, 2016 ▸)

	Findable
F1	(Meta)data are assigned a globally unique and persistent identifier
F2	Data are described with rich metadata (defined by R1 below)
F3	Metadata clearly and explicitly include the identifier of the data they describe
F4	(Meta)data are registered or indexed in a searchable resource

	Accessible
A1	(Meta)data are retrievable by their identifier using a standardized communications protocol
A1.1	The protocol is open, free and universally implemented
A1.2	The protocol allows for an authentification and authorization procedure, where necessary
A2	Metadata are accessible, even when the data are no longer available

	Interoperable
I1	(Meta)data use a formal, accessible, shared and broadly applicable language for knowledge representation
I2	(Meta)data use vocabularies that follow FAIR principles
I3	(Meta)data include qualified references to other (meta)data

	Reusable
R1	Meta(data) are richly described with a plurality of accurate and relevant attributes
R1.1	(Meta)data are released with a clear and accessible data usage licence
R1.2	(Meta)data are associated with detailed provenance
R1.3	(Meta)data meet domain-relevant community standards
